# Chemoprevention of oxidative stress-associated oral carcinogenesis by sulforaphane depends on NRF2 and the isothiocyanate moiety

**DOI:** 10.18632/oncotarget.10609

**Published:** 2016-07-15

**Authors:** Aixian Lan, Wenjun Li, Yao Liu, Zhaohui Xiong, Xinyan Zhang, Shanshan Zhou, Olesya Palko, Hao Chen, Mayanga Kapita, Justin R. Prigge, Edward E. Schmidt, Xin Chen, Zheng Sun, Xiaoxin Luke Chen

**Affiliations:** ^1^ Department of Oral Medicine, Beijing Stomatological Hospital & School of Stomatology, Capital Medical University, Beijing 100050, China; ^2^ Cancer Research Program, JLC-BBRI, North Carolina Central University, Durham, NC 27707, USA; ^3^ Department of Pharmaceutical Engineering, School of Pharmaceutical & Life Sciences, Changzhou University, Jiangsu 213164, China; ^4^ Department of Immunology and Infectious Diseases, Montana State University, Bozeman, MT 59717, USA

**Keywords:** oral cancer, chemoprevention, NRF2, sulforaphane, 4NQO

## Abstract

Oxidative stress is known to play an important role in oral cancer development. In this study we aimed to examine whether a chemical activator of NRF2, sulforaphane (SFN), may have chemopreventive effects on oxidative stress-associated oral carcinogenesis. We first showed that Nrf2 activation and oxidative damage were commonly seen in human samples of oral leukoplakia. With gene microarray and immunostaining, we found 4-nitroquinoline 1-oxide (4NQO) in drink activated the Nrf2 pathway and produced oxidative damage in mouse tongue. Meanwhile whole exome sequencing of mouse tongue identified mutations consistent with 4NQO's mutagenic profile. Using cultured human oral keratinocytes and 4NQO-treated mouse tongue, we found that SFN pre-treatment activated the NRF2 pathway and inhibited oxidative damage both *in vitro* and *in vivo*. On the contrary, a structural analogue of SFN without the isothiocyanate moiety did not have such effects. In a long-term chemoprevention study using wild-type and *Nrf2*^-/-^ mice, we showed that topical application of SFN activated the NRF2 pathway, inhibited oxidative damage, and prevented 4NQO-induced oral carcinogenesis in an Nrf2-dependent manner. Our data clearly demonstrate that SFN has chemopreventive effects on oxidative stress-associated oral carcinogenesis, and such effects depend on *Nrf2* and the isothiocyanate moiety.

## INTRODUCTION

Oral cancer is one of the major public health problems worldwide, as well as a major cause of cancer morbidity and mortality. In the United States, approximately 30,260 new cases are estimated and 5,990 cases may die in 2015 [1]. Oral squamous cell carcinoma (SCC) is the most common type of oral cancer, which usually develops from precancerous lesions such as oral leukoplakia (OLK) and erythroplakia, and histopathologically follows a step-wise pattern of hyperplasia, dysplasia and SCC [2]. Overall survival of these patients remained unchanged despite the advances in radiotherapy and chemotherapy. The five-year survival rate for patients with early and localized lesions is ~80%, whereas it is only 19% for patients with distant metastasis [3]. Thus it is important to develop preventive strategies for this deadly disease.

Oxidative stress has long been known to play an important role in the development of human oral cancer. 8-oxo-2′-deoxyguanosine (8OHdG), a marker of DNA oxidative damage, is known to increase in OLK and SCC as compared with normal mucosa [4–6]. Major risk factors of oral cancer (tobacco, alcohol drinking, and betel nut chewing) all generate oxidative stress in the epithelial cells of oral mucosa [7]. Although antioxidants have been proposed and tested for oral cancer prevention, randomized controlled clinical trials did not provide evidence that dietary antioxidant supplements are beneficial in primary cancer prevention. A systematic review conducted for the United States Preventive Services Task Force failed to find clear benefits of vitamin and mineral supplements for the prevention of chronic diseases including cancer [8]. Thus activation of the antioxidative stress response by chemicals (indirect antioxidants) has become a reasonable and promising approach.

As a major cellular defense pathway, nuclear factor erythroid 2-like 2 (NRF2) is known to regulate expression of genes involved in detoxification and anti-oxidative stress response. NRF2 forms heterodimers with small MAF proteins and binds to the antioxidant response elements of target genes when cells are exposed to oxidative stress or xenobiotics. Kelch-like ECH-associated protein 1 (KEAP1) inhibits the function of NRF2 by retaining NRF2 in the cytoplasm under normal physiological conditions, and by allowing nuclear translocation of NRF2 under stress conditions [9]. Paradoxically, NRF2 is known to be overexpressed in human oral cancer suggesting the involvement of NRF2 in oral carcinogenesis [10, 11].

In this study, we aimed to elucidate the chemopreventive effect and mechanisms of a chemical activator of NRF2 on oxidative stress-associated oral carcinogenesis. Sulforaphane (SFN) is a compound extracted from broccoli. Its isothiocyanate moiety (-N=C=S) is highly electrophilic and reacts directly but reversibly with the sulfhydryl groups of specific cysteine residues of Keap1, especially Cys^151^. Disruption of KEAP1-NRF2 interactions by SFN leads to nuclear accumulation of NRF2 and up-regulation of its target genes [12]. The 4NQO-induced oral carcinogenesis model in mice was used to test the chemopreventive efficacy and mechanisms of SFN *in vivo* [13, 14]. Cultured human cells and human tissue samples were also employed to understand chemopreventive mechanisms and establish clinical relevance.

## RESULTS

### Oxidative damage and NRF2 activation in human oral leukoplakia

**Figure 1:**
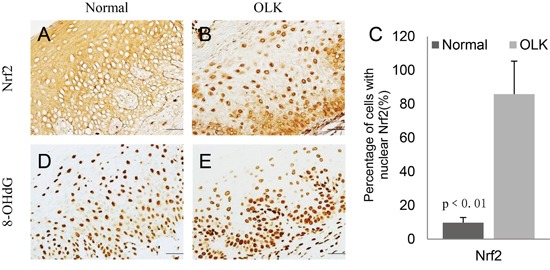
Increased expression of Nrf2 and 8OHdG in the nuclei of oral epithelial cells in OLK patients as compared with normal subjects **A-D.** IHC staining of NRF2 and 8OHdG in normal and OLK samples; **E.** Quantitation showed a significant increase of the number of epithelial cells with nuclear NRF2 in OLK samples as compare with normal samples.

In OLK samples, NRF2 was activated as evidenced by its nuclear accumulation (Figure 1A, 1B). The number of epithelial cells with nuclear NRF2 was significantly higher in OLK than in the normal oral mucosa (Figure 1C). As a marker of oxidative DNA damage, 8OHdG was increased in OLK (Figure 1D, 1E).

### 4NQO produces oxidative DNA damage and gene mutations, and activates the Nrf2 pathway in mouse tongue

With gene microarray analysis and a stringent data analysis method, we found 32 genes up-regulated and one gene down-regulated in mouse tongue due to 4NQO treatment (100μg/ml in drink for 8 weeks) as compared to control. Among these 33 genes, 9 are known Nrf2-regulated genes (*Slc7a11, Akr1b8, Gsta1/a2, Gclc, Ptgr1, Gsta4, Gsta3, Gpx2, Ftl1/Ftl2*). Several other genes (*Brca1, Mcm5, Mcm3, Slc2a1, Clspn, Ucp3*) are functionally associated with NRF2 or oxidative stress response [15–21] (Suppl. Excel 1). GSA_CP analysis showed that 58 canonical pathways were enriched in 4NQO-treated samples. These pathways mainly belonged to three categories: (1) 10 gene sets associated with DNA damage repair, such as nuclear excision repair, mismatch repair, DNA repair, p53 signaling pathway, ATM pathway; (2) 28 gene sets associated with cell cycle and DNA replication; (3) 6 gene sets associated with stress response and detoxification, such as glutathione metabolism, metabolism of xenobiotics, drug metabolism, biological oxidations, glutathione conjugation, Phase II conjugations (Suppl. Excel 2). In addition, an NRF2-regulated gene set, was also enriched in 4NQO-treated samples (Suppl. Excel 3).

**Figure 2:**
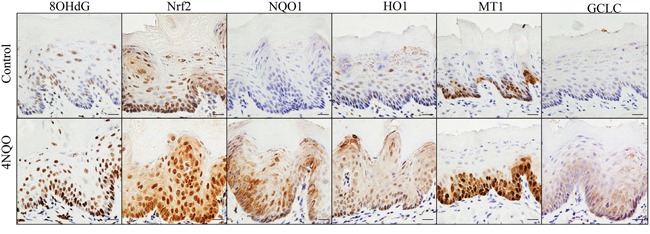
4NQO treatment enhances oxidative DNA damage (8OHdG) and up-regulates expression of NRF2 and its target genes in mouse tongue (NQO1, HO1, MT1 and GCLC)

IHC was performed to compare expression of 8OHdG, NRF2, and four classical NRF2-regulated genes (NQO1, HO1, MT1 and GCLC) in 4NQO-treated tongue and control tongue. All these markers were dramatically overexpressed in the 4NQO-treated tongue epithelial cells (Figure 2). We further analyzed DNA mutations in 4NQO-treated tongue using whole exome sequencing. Single nucleotide variants (SNVs) mainly took place at the G nucleotide. In sample 4NQO1, 28 out of 75 single SNVs took place at the G nucleotides. In sample 4NQO2, 54 out of 153 SNVs took place at the G nucleotides (Suppl. Excel 4). The preference for mutations at the G nucleotide is consistent with a recent whole genome sequencing study on the mutagenic spectrum of 4NQO in *Aspergillus nidulans* [22]. These data clearly demonstrated that 4NQO treatment results in oxidative damage, DNA mutations and Nrf2 activation in squamous epithelial cells of mouse tongue, and also suggested that the 4NQO-induced oral carcinogenesis model in mice is a relevant model system for studies on oxidative stress-associated oral cancer.

### SFN activates Nrf2 in oral squamous epithelial cells and prevents 4NQO-induced oxidative damage *in vitro* and *in vivo*

**Figure 3:**
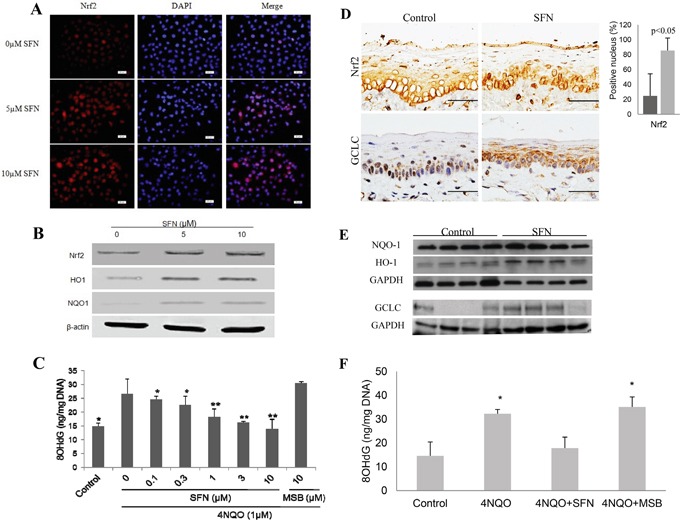
SFN treatment up-regulates NRF2 and its target genes, and suppresses 4NQO-induced oxidative DNA damage *in vitro* and *in vivo* **A.** There is a dose-dependent increase of nuclear NRF2 in DOK cells after SFN treatment as determined by immunofluoresent staining with DAPI counterstaining. **B.** SFN treatment up-regulates expression of NRF2, HO1 and NQO1 in DOK cells as determined by Western blotting. **C.** Pre-treatment of SFN inhibits the level of 8OHdG after 4NQO treatment of DOK cells, whereas MSB does not have such an effect. **D.** IHC and quantitation shows a significant increase of the number of epithelial cells with nuclear Nrf2 and an increase of GCLC expression in mouse tongue after topical SFN treatment. **E.** Western blotting of NQO1, HO1 and GCLC shows a significant increase in HO1 (p<0.05) and slight increase of NQO1 and GCLC in SFN-treated mouse tongue. **F.** SFN treatment reduces the level of 8OHdG in mouse tongue due to 4NQO treatment. MSB does not have such an effect.

SFN was examined for its activating effect on Nrf2 and inhibitory effect on oxidative DNA damage *in vitro* and *in vivo*. SFN treatment of DOK cells not only increased NRF2 expression, but also its nuclear accumulation (Figure 3A). Western blotting confirmed that SFN up-regulated the expression of NRF2 and its target genes (HO1 and NQO1) in a dose-dependent manner (Figure 3B). More importantly, treatment of DOK cells with SFN prior to 4NQO treatment prevented 4NQO-induced oxidative DNA damage in a dose-dependent manner. On the contrary, MSB, a SFN analogue without the isothiocyanate moiety, did not have any protective effect on oxidative DNA damage (Figure 3C).

Topical application of SFN on mouse tongue for 2 weeks induced nuclear accumulation of NRF2 and up-regulation of GCLC in squamous epithelial cells of mouse tongue. Semi-quantitation of NRF2 staining showed a significant increase of epithelial cells with nuclear NRF2 in SFN-treated samples (Figure 3D). Western blotting also showed up-regulation of HO1, NQO1 and GCLC by SFN (Figure 3E). As expected, topical application of SFN for two weeks prior to 4NQO treatment inhibited oxidative DNA damage. In contrast, topical application of MSB did not have a significant effect on 4NQO-induced oxidative DNA damage (Figure 3F).

### SFN prevents 4NQO-induced oral carcinogenesis in wild-type mice, but not in *Nrf2*^-/-^ mice

In a long-term experiment, SFN were topically applied to tongues of wild-type and *Nrf2*^-/-^ mice for 25 weeks (1 week prior to 4NQO treatment, 16 weeks during 4NQO treatment, and 8 weeks afterwards). SFN significantly reduced the number of visible tumors and the incidence of tongue SCC in wild-type mice. However the chemopreventive effect of SFN was completely lost in *Nrf2*^-/-^ mice (Table 1).

**Table 1:** Chemopreventive effects of sulforaphane on 4NQO-induced oral carcinogenesis ^a^

Group	Genotype	Treatment	No	Visible Tumor (%)	No. of Tumor	Tumor Volume (mm^3^)	Dysplasia	SCC
A	Wild-type	-	10	-	-	-	-	-
B	Nrf2^-/-^	-	10	-	-	-	-	-
C	Wild-type	4NQO	26	21 (80.8%)	1.00±0.63	5.27±10.09	8(30.8%)	18(69.2%)
D	Wild-type	4NQO, SFN (-1~24w)	28	14 (50%)^b^	0.61±0.69	5.80±13.90	17(60.7%)	11(39.3%)^b^
E	Nrf2^-/-^	4NQO, SFN (-1~24w)	24	17 (70.8%)	1.42±1.21^c^	5.35±9.01	8(33.3%)	16(66.7%)^d^

a 4NQO was given in drinking water (50μg/ml) for 16 weeks, and then replaced by water for 8 weeks. SFN was topically applied on the tongue at the concentration of 50 mM (50μl, 3 times per week).

b *P*<0.05 (as compared with Group C)

c *P*<0.01 (as compared with Group D)

d *P*<0.05 (as compared with Group D)

**Figure 4:**
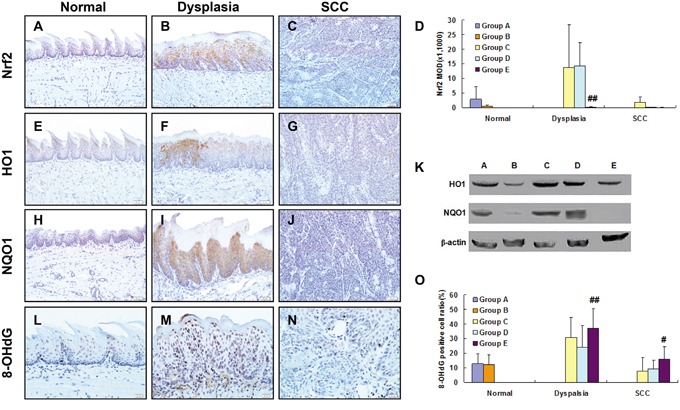
Expression of NRF2, HO1, NQO1 and 8OHdG in 4NQO/SFN-treated mouse tongue: Normal epithelium (A, E, H, L), dysplasia (B, F, I, M) and SCC (C, G, J, N) Expression of NRF2 in the epithelial cells oral tissues was semi-quantitated after (D). ^##^ Statistically different from Group C (p<0.01). Expression of HO1 and NQO1 as determined by Western blotting (K). DNA oxidative damage level (the percentage of 8-OHdG positive cells) in the epithelial cells was semi-quantitated after IHC staining (O). ^#^ Statistically different from Group C (p<0.05); ^##^ Statistically different from Group C (p<0.01).

With IHC staining, we examined expression of Nrf2, HO1, NQO1 and 8OHdG in mouse tongue. Increased expression of NRF2 was observed in dysplasia but not in SCC as compared with normal samples (Figure 4A, 4B, 4C). Semi-quantitation of NRF2 expression confirmed this expression pattern (Figure 4D). Using IHC and Western, we also examined expression of HO1 and NQO1. Both NRF2-regulated genes shared the same expression pattern of NRF2 in normal, dysplasia and SCC tissues (Figure 4E-4J; semiquantiatation data of HO1 and NQO1 expression are not shown). Topical treatment with SFN up-regulated expression of HO1 and NQO1 in wild-type tongue (Group D), but not in *Nrf2*^-/-^ tongue (Group E) (Figure 4K). Consistent with this observation, 8OHdG was at the highest level in dysplasia, and slightly reduced by SFN in wild-type tongue, but not in *Nrf2*^-/-^ tongue (Figure 4O).

## DISCUSSION

In this study, SFN was shown to prevent 4NQO-induced oxidative damage and oral carcinogenesis in mouse tongue. Both pharmacological and genetic approaches demonstrated that SFN exerted its chemopreventive effects through NRF2 and the isothiocyanate moiety *in vitro* and *in vivo*. Using cell culture, mouse model, and human samples, we validated SFN as an effective chemopreventive agent for oxidative stress-associated oral carcinogenesis.

**Figure 5:**
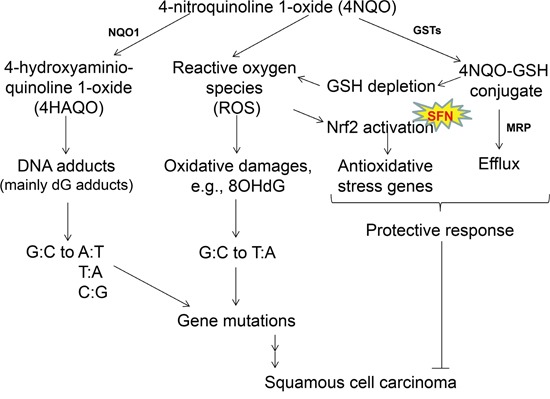
Mechanisms of 4NQO-induced oral carcinogenesis, and the chemopreventive mechanisms of SFN When 4NQO is quickly metabolized in the squamous epithelial cells by NQO1, the ultimate carcinogen (4-hydroxyaminoquinoline 1-oxide, 4HAQO) causes gene mutations. Meanwhile, metabolism by glutathione transferases (GSTs) results in glutathione (GSH) depletion in these cells as well, and generates ROS which also produces oxidative damages and gene mutations. These gene mutations are presumably the driving force leading to SCC in mouse tongue. As a protective response, 4NQO-GSH conjugate is expected to be eliminated through multidrug resistant protein (MRP), and ROS activates the Nrf2 pathway which regulates antioxidative stress genes such as MT1, HO1, superoxide dismutase (SOD) and catalase. It is known that NQO1, GSTs, MRP, SOD, catalase are all transcriptionally regulated by NRF2. Based on this proposed mechanism, the 4NQO-induced oral carcinogenesis model in mice shares the same mechanism of oxidative damage as human oral carcinogenesis. SFN, which acts as a chemopreventive agent for 4NQO-induced oral carcinogenesis in mouse tongue through NRF2 and the isothiocyanate moiety, is therefore potentially effective for chemoprevention of human oral cancer.

The 4NQO-induced oral carcinogenesis model with mice has been widely used for mechanistic and chemopreventive studies on oral cancer [13, 14]. It is known that 4NQO produces oxidative stress directly through generating reactive oxygen species (ROS) and indirectly through depleting GSH, and thus generates oxidative DNA damages [23, 24]. 4NQO also induced the formation of cellular topoisomerase I-DNA cleavage complexes, which may contribute to its mutagenesis and carcinogenesis [25]. Our microarray data of 4NQO-treated mouse tongue confirmed NRF2 activation and up-regulation of NRF2-regulated genes as oxidative stress response (Figure 2; Suppl. Excel 1, 2, 3). These data are consistent with a RNA-seq study comparing control mouse tongue with 4NQO-treated mouse tongue. In addition to up-regulation of 375 genes and down-regulation of 310 genes in that study, pathway analysis showed an enrichment of gene sets like “response to oxidative stress, glutathione metabolism, metabolism of xenobiotics by cytochrome P450” [26]. These data support the notion that 4NQO-induced oral carcinogenesis in mice (Figure 5) mimics oral carcinogenesis in humans at least in oxidative stress-associated mechanisms. It should be noted that exome sequencing data of 4NQO-treated mice showed some differences between mouse model and human cancer. In human oral cancer, C:G>A:T transversion and mutations at C nucleotides are more commonly seen [27, 28]. In this study, we also found that NRF2 activation was more prominent at the stage of dysplasia than the stage of cancer (Figure 4). These differences suggest multiple etiological factors for human oral cancer apart from oxidative stress.

The NRF2 pathway is a major cellular defense pathway through transcriptional regulation of its target genes involved in detoxification and anti-oxidative stress response. For example, NQO1 is a widely-distributed FAD-dependent flavoprotein that promotes obligatory 2-electron reductions of quinones, quinoneimines, nitroaromatics, and azo dyes. By reducing quinone levels, NQO1 minimizes reactive oxygen intermediates and prevents depletion of intracellular thiol pools [29]. HO1 is an oxidative stress-inducible protein which catabolizes heme into carbon monoxide, biliverdin/bilirubin and free iron, and thereby maintains intracellular redox homeostasis [30]. As a small, cysteine-rich and heavy metal-binding protein, MT1 protects cells from oxidative stress [31]. GCLC is the catalytic unit of glutamate cysteine ligase which catalyzes the rate-limiting step in the formation of the cellular antioxidant glutathione [32]. That's why *Nrf2*^-/-^ mice were more susceptible to 4NQO-induced tongue and esophageal carcinogenesis than wild-type mice, whereas *Keap1*-knockdown mice were resistant [33]. Thus, chemical activators of NRF2 are anticipated to be chemopreventive against oxidative stress-associated carcinogenesis. Such chemicals are classified into about ten chemically distinct classes based on their chemical structures and the nature of reaction with cysteine sulfhydryl groups [34]. Irrespective of their chemical differences, many of these compounds have shown chemopreventive effects against 4NQO-induced mutagenesis or carcinogenesis, such as green tea polyphenols [35], lycopene [36], selenium and vitamin E [37], butylated hydroxyanisole [38], naturally occurring plant phenolics [39], dietary flavonoids chalcone [40], naturally occurring xanthophylls [41], curcumin [42], and indole-3-carbinol [43].

SFN was chosen as a candidate chemopreventive agent in this preclinical study due to its advantages in multiple aspects. First of all, SFN is quite potent in activating NRF2 when topically applied on the skin [44, 45]. Indeed, SFN is ranked at the top among the natural NRF2 activators [46]. Secondly, SFN is a small molecule compound (MW 177) and stable in the polyethylene glycol based formulation suitable for topical application [47]. Thirdly, as a compound within the isothiocyanate group of organosulfur compounds, SFN is particularly rich in young cruciferous vegetables such as broccoli, Brussels sprouts or cabbages. It is produced when the enzyme myrosinase transforms glucoraphanin upon damage to the plant. SFN can be administered to the oral mucosa not only through topical application of the pure compound, but also through chewing on SFN-rich broccoli sprouts. Both the natural source and synthetic form of SFN are inexpensive and thus would allow the long-term use for oral cancer prevention. Fourthly, SFN has been used in clinical trials for human diseases with very good safety profiles [48].

There are two major concerns of using SFN for oral cancer prevention. NRF2 is a double-edged sword and can be carcinogenic when overactive. *Nrf2* was found to prevent inhibition but accelerate progression of lung carcinogenesis *in vivo* [49]. *K-Ras(G12D), B-Raf(V619E)* and *Myc(ERT2)* each increased the transcription of *Nrf2* to confer a more reduced intracellular environment. Genetic targeting of the *Nrf2* pathway impairs *K-Ras(G12D)*-induced proliferation and tumorigenesis *in vivo* [50]. These data suggest that careful timing of NRF2 activation is a critical issue in balancing the induction and prevention of oral carcinogenesis. Although there is a need to induce NRF2 before cancer initiation, further activation of NRF2 at certain late stage may promote carcinogenesis. To reconcile the good and bad sides of NRF2, Kensler and Wakabayashi proposed a concept of “inflection point” to explain why intermittent dosing with an NRF2 activator (e.g., one or several doses a day) is unlikely to promote carcinogenesis in contrast to constitutive NRF2 activation when *Nrf2* or *Keap1* is mutated [51]. In fact, SFN acts on KEAP1 in a reversible manner. As a result, systemic administration of SFN did not promote the growth of *K-ras(G12D)*-induced lung tumors and had no significant effect on the growth of established tumor xenografts in nude mice [52]. Therefore we do not expect topical application of SFN would promote oral carcinogenesis. It is also possible that SFN may exert its chemopreventive effect through NRF2-independent mechanisms, such as cell cycle regulation, induction of apoptosis, etc [53, 54]. Since these NRF2-independent effects usually take place at high concentrations and the chemopreventive effect of SFN was diminished in *Nrf2*^-/-^ mice, it is therefore believed that SFN mainly acts through NRF2 in this current study.

While this manuscript was in preparation, one abstract was published to evaluate the chemopreventive efficacy and mechanisms of NRF2 for oral carcinogenesis *in vitro* and *in vivo*. It is very interesting that short-term treatment of healthy volunteers with topical or oral SFN-rich broccoli sprout extract resulted in up-regulation of NQO1 expression in oral mucosa of 70% subjects (7 out of 10) [55].

In summary, these data clearly demonstrate that SFN has chemopreventive effects on oxidative stress-associated oral carcinogenesis, and such chemopreventive effects depend on NRF2 and the isothiocyanate moiety. Clinical chemoprevention trials using topical SFN in humans are therefore warranted.

## MATERIALS AND METHODS

### Collection of human samples

A total of 15 samples of normal epithelium and 21 samples of OLK were collected from human patients in the Department of Oral Medicine, Beijing Stomatological Hospital, Capital Medical University, from 2012 to 2014. The informed consent was obtained from all patients. The OLK lesion and normal mucosa were biopsied, fixed in 10% buffered formalin and embedded in paraffin. OLK was defined as a white patch or plaque that cannot be characterized clinically or pathologically as any other disease [56].

### Synthesis of sulforaphane (SFN) and 4-methylsufinylbutylamine (MSB)

**Figure 6:**
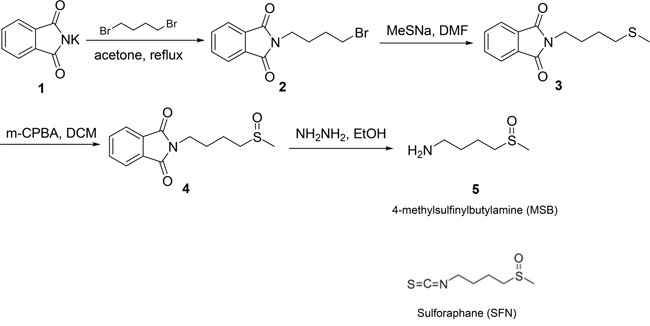
Chemical structures of SFN and MSB, and the synthetic process of MSB

SFN is known to activate NRF2 through a reaction between its isothiocyanate moiety with Keap1 cysteine sulfhydryl group [57]. SFN was synthesized by us (SZ and XC) using a previously published method of chemical synthesis [58]. MSB was synthesized as a SFN analogue without the isothiocyanate moiety (Figure 6). The detailed procedure of chemical synthesis and MRI data are provided in Supplementary File.

### Cell culture and treatment

Human dysplastic oral keratinocytes (DOK) were obtained from Sigma (St Louis, MO). The DOK cells were cultured with DMEM/High glucose (Hyclone) medium supplemented with 15% FBS in culture incubator at 37C in 5% CO2. To determine whether SFN treatment may activate Nrf2 pathway, we determined Nrf2 localization with immunofluorescent staining. A total of 5×10^4^ cells were cultured on a 24-well plate and treated with SFN (5 or 10μM) for 24 hours. Cells were fixed in cold 10% buffered neutral formalin for 10 minutes for immunofluorescent staining of NRF2.

To determine whether SFN treatment may inhibit 4NQO-induced oxidative damage, cells were treated by SFN (0.1, 0.3, 1, 3, or 10μM) or MSB (10μM) for 24 hr, and then 4NQO (1μM) for 12hr. These concentrations were determined in a preliminary experiment in which SFN and MSB (≤10μM) and 4NQO (≤1μM) did not significantly suppress cell growth as determined by a cell proliferation assay (data not shown). The cells were harvested for measurement of 8-OHdG by enzyme immunoassay (EIA).

### Immunohistochemical staining (IHC) and immunofluorescent staining

Human samples of OLK lesion and normal mucosa were stained with a rabbit anti-NRF2 antibody (1:200) [59] or anti-8-oxo-2′-deoxyguanosine (8OHdG, 1:800; Abcam, Cambridge, MA). After deparaffinization, sections were submerged in methanol containing 0.3% hydrogen peroxide for 15 min to inhibit endogenous peroxidase activity. Antigen retrieval was done for incubating the sections in 0.01 mol/L citrate buffer (pH 6) in microwave. The antigen was detected with astreptavidin-peroxidase reaction kit (VECTASTAIN Elite ABC Kit; Vector Labs, Burlingame, CA). To ensure the specificity of the primary antibody, control tissue sections were incubated in the absence of primary antibody.

Immunofluorescent staining of NRF2 was conducted on cells with a rabbit polyclonal anti-NRF2 antibody (1:400), and then a goat anti-rabbit IgG secondary antibody labeled with Alexa Fluor 546 (Invitrogen, Carlsbad, CA). After counterstaining with DAPI, fluorescent signals were visualized under a fluorescent microscope.

Mouse tissue sections were analyzed for 8OHdG and expression of NRF2, NAD(P)H dehydrogenase [quinone] 1 (NQO1), heme oxygenase 1 (HO1), metallothionein 1 (MT1), and glutamate-cysteine ligase, catalytic subunit (GCLC) by IHC with the following antibodies overnight at 4C: a rabbit polyclonal anti-8-OHdG antibody (1:8000; Abcam), a rabbit polyclonal anti-NRF2 (1:200) [59], a rabbit polyclonal anti-NQO1 antibody (1:8000; Abcam), a mouse monoclonal anti-MT1 (1:100; Accurate Chemical & Scientific, Westbury, NY), a rabbit polyclonal anti-HO1 (1:25; Abcam), and a rabbit polyclonal anti-GCLC (1:100; LifeSpan BioSciences, Seattle, WA).

Immunostaining was semiquantitated for comparison between groups in some experiments. Expression of NRF2 was semi-quantitated using a computerized image analysis system (ImagePro Plus; Media Cybernetics, Rockville, MD). The area of positive staining and the mean optical density were measured for calculation of integrated optical density (IOD) by multiplying density by area. The percentage of 8OHdG-positive cells was calculated as the number of positively stained cells divided by the total number of tongue epithelial cells. Three non-contiguous, randomly selected, high-power fields (x400) were photographed and counted per sample.

### Western blotting

Cells were treated with 5 or 10μM SFN for 24 hours, lysed and fractionated to obtain cytoplasmic and nuclear fractions. The nuclear extract was used for Western blotting of NRF2, and the cytoplasmic extract for HO1 and NQO1. Western blotting was done with a standard protocol. In brief, 20μg of protein was resolved on a SDS-polyacrylamide gel and transferred to nitrocellulose membranes. NRF2, HO1, and NQO1 were detected with a rabbit polyclonal anti-NRF2 antibody (1:1000; Abcam), a rabbit polyclonal anti-HO1 antibody (1:2000; Abcam), and a mouse polyclonal anti-NQO1 antibody (1:1000; Cell Signaling, Danvers, MA), respectively. Membranes were developed with ECL chemiluminescence and exposed on X-ray film. Quantitation of the bands on the film was carried out by a densitometer. Mouse tongue samples were homogenized and lysed in RIPA buffer for Western blotting of NQO1, HO1 and GCLC in a similar way. GAPDH or β-actin was detected as a loading control.

### Enzyme immunoassay (EIA) for 8-OHdG

The cells were lysed in 100μl buffer (25 mM NaOH, 0.2 mM EDTA) at 95C for 60 min. After DNA extraction, 8OHdG was measured using 8OHdG ELISA Kit (Rapidbio, West Hills, CA) according to the manufacturer's instructions. The level of 8OHdG was expressed as ng per mg total DNA. Mouse tongue was weighed and homogenized, lysed in the buffer, DNA extracted, and measured for 8-OHdG in a similar way.

### Affymetrix gene microarray and data analysis

All the experimental procedures involving the use of mice were approved by the Institutional Animal Use and Care Committee, Beijing Stomatological Hospital. C57BL/6J mice were given 4NQO (100μg/ml in drink) for 8 weeks. Non-treated control samples were used for comparison. Total RNA was extracted from individual mouse tongue tissue using TRIzol (Invitrogen, Carlsbad, CA). These RNA samples were checked for their quality using gel electrophoresis and their concentrations were measured using spectrophotometry. GeneChip mouse Genome Array (Affymetrix, Santa Clara, CA) containing 45,101 oligo probes were used to detect differential gene expression between tongue tissues of control and treated mice. One μg of total RNA was used for labeling and processing after quality validation. GeneChip hybridization and scanning were performed according to the Affymetrix protocols. Briefly, double-stranded cDNA containing T7 promoter (Genset, La Jolla, CA) was synthesized from total RNA using the SuperScript Choice System (Invitrogen). Biotinylated cRNAs were generated from cDNAs by *in vitro* transcription and amplified by using the BioArray T7 RNA polymerase labeling kit (Enzo Diagnostics). After purification of cRNAs by GeneChip Sample Cleanup Module (Affymetrix), 15 μg of cRNA was fragmented at 94°C for 35 min. Approximately 12.5 μg of fragmented cRNA was used in a 250-μl hybridization mixture containing herring-sperm DNA (0.1 mg/ml; Promega), plus bacterial and phage cRNA controls (1.5 pM BioB, 5 pM BioC, 25 pM BioD, and 100 pM Cre) to serve as internal controls for hybridization efficiency. Aliquots (200 μl) of the mixture were hybridized to arrays for 16 h at 45°C in a GeneChip Hybridization Oven 640 (Affymetrix). Each array was washed and stained with streptavidin–phycoerythrin (Invitrogen) and amplified with biotinylated anti-streptavidin antibody (Vector Laboratories) on the GeneChip Fluidics Station 450 (Affymetrix). Arrays were scanned with the GeneArray G7 scanner (Affymetrix) to obtain image and signal intensities. Data pre-processing was carried out in R with Bioconductor package biocLite for quality filtering and data normalization. All probe sequences were BLAT against Affymetrix mouse annotation (Mouse430_2.na32.annot.csv), and were annotated with Gene Symbol.

Significance analysis of microarrays (SAM) was used for identification of differentially expressed genes. Pre-processed data were used to construct a series of data matrix files for further analysis. For a given data matrix, the rows were excluded if more than 40% of missing values were observed. The rest of missing data was imputed with a K-nearest neighbor (k=9) approach. Differentially expressed genes were obtained from two-class SAM in Excel with the median number of false positive less than 1. Gene set analysis (GSA) was carried out as an add-in in Excel to identify differentially expressed gene sets. 1,000 permutations were applied to generate a null distribution for statistical testing, and significantly enriched gene sets were obtained at a false discovery rate cutoff of 0.5. Curated gene sets in canonical pathway (CP; 880 gene sets) were downloaded from the GSEA web portal and used in this study (http://www.broadinstitute.org/gsea/index.jsp). In addition, we generated an NRF2-regulated gene set based on our previous gene array study [60] (Suppl. Excel 3), and conducted GSA accordingly. The microarray data have been submitted to the GEO database (GSE39629).

### Whole exome sequencing

DNA samples of 3 mouse tongues were analyzed by whole exome sequencing: one control, one 4NQO-treated (100μg/ml in drink for 8 weeks) and one 4NQO plus ethanol treated (100μg/ml 4NQO and 8% ethanol in drink for 8 weeks). Genomic DNA was extracted from mouse tongue epithelium, quality checked, and submitted to Otogenetics Corporation (Norcross, GA) for exome capture and sequencing. Briefly, genomic DNA was subjected agarose gel and OD ratio tests to confirm the purity and concentration prior to Covaris (Covaris, Inc., Woburn, MA) fragmentation. Fragmented DNAs were tested for size distribution and concentration using an Agilent Bioanalyzer 2100 and Nanodrop. Illumina libraries were made from qualified fragmented DNA using NEBNext reagents (New England Biolabs, Ipswich, MA) and the resulting libraries were subjected to exome enrichment using SureSelect XT Mouse All Exon Kit (Agilent Technologies, Wilmington, DE) following manufacturer's instructions. Enriched libraries were tested for enrichment by qPCR and for size distribution and concentration by an Agilent Bioanalyzer 2100. The samples were then sequenced on an Illumina HiSeq2000 which generated paired-end reads of 100 nucleotides (nt). Data was analyzed for data quality, exome coverage, and exome-wide SNP/InDel using the platform provided by DNAnexus (DNAnexus, Inc, Mountain View, CA). This method targets 49.6 Mb of mouse exome. Exon definition is derived from Ensembl and RefSeq, designed against mm9 reference from UCSC. Gene mutations were analyzed by comparing data of the control tongue with that of 4NQO-treated tongue (one treated with 4NQO alone and another with 4NQO and ethanol) after noncoding and synchronymous SNVs were removed. The minimum depth of sequencing was set at 10.

### Animal experiments with SFN

In order to determine whether topical SFN may activate NRF2 in mouse tongue, a two-week study was performed in which C57BL/6J mice (6-8 weeks old; n=5; Beijing Vital River Laboratory Animal Company, Beijing, China) were topically treated with SFN (100 mM, 50μl, 5 times per week). In the literature, 0.1-1 μmole per day have been used for topical application on mouse skin for preventing skin cancer and treating pachyonychia congenital [44, 61]. We chose 5 μmole per day because this study was a proof-of-concept experiment which will be followed by a dose-response experiment. Unlike topical application on mouse skin, topical application into mouse oral cavity may be less bioavailable because mice tended to spit excessive liquid right after application. A control group of mice (n=5) were treated in the same way with corn oil. At the end of the study, half of the tongue was collected and fixed in 10% buffered formalin and embedded in paraffin for IHC of NRF2 and GCLC. The second half of the tongue was snap-frozen in liquid nitrogen and stored at -80C for Western blotting of NQO1, HO1, and GCLC.

In order to determine whether topical SFN may protect mouse tongue from 4NQO-induced oxidative damage, wild-type C57BL/6J mice (6-8 weeks old) were treated with 4NQO in drinking water (100μg/ml) for 8 weeks. Two weeks before and during 4NQO treatment, the first group did not receive any other treatment (n=15); the second group was topically treated with SFN (n=15; 100 mM, 50μl, 5/week); and the third group with MSB (n=15; 100 mM, 50μl, 5/week). Mice not treated with 4NQO (n=10) served as the negative control. All the mice were sacrificed at the end of week 10. One half of the tongue was snap-frozen in liquid nitrogen for analysis of 8-OHdG and the other half was fixed in formalin and embedded in paraffin.

In a long-term chemoprevention study with SFN, wild-type C57BL/6J mice (6-8 weeks old) and *Nrf2*^-/-^ mice (RIKEN BioResource Center, Japan) were bred in-house and PCR genotyped [62]. Mice were assigned to 5 groups: Group A (n=10) as the wild-type negative control; Group B (n=10) as the Nrf2^-/-^ negative control; Group C (n=30) as the positive control group; Group D (n=30) as the wild-type experimental group; and Group E (n=30) as the *Nrf2*^-/-^ experimental group. Group C, D and E were given 4NQO in drinking water (50μg/ml for 16 weeks). SFN treatment (50 mM in corn oil, 50μl, 3/week) were administered to Group D and E throughout the whole experiment (-1~24w) (Table 1). An earlier study has clearly demonstrated that *Nrf2^-/-^* mice were more susceptible to 4NQO-induced tongue carcinogenesis than wild-type mice [33]. Therefore, we did not include an “*Nrf2^-/-^*+4NQO” group and an “*Nrf2^-/-^*+4NQO+ SFN” group for the sake of saving the number of animals. All mice were monitored for their body weights weekly and sacrificed at Week 24. The tongue was harvested and examined for the presence of macroscopic alterations, then split longitudinally. One half of the tongue was snap frozen in liquid nitrogen for Western blotting (HO1 and NQO1), and the half was fixed overnight in 10% neutral-buffered formalin, processed, and embedded in paraffin for histopathology.

Histopathological analysis was performed blind by our research pathologist without prior knowledge of the experiment. Dysplasia and SCC were diagnosed with established criteria. Dysplasia was characterized by irregular epithelial stratification, increased number of mitotic figures, increased nuclear-to-cytoplastic ratio, and loss of polarity of basal cells. SCC was diagnosed when dysplastic cells invaded underlying tissues [56]. Tissue sections were used for analysis of Nrf2, HO1, NQO1, and 8-OHdG with IHC.

### Statistical analysis

Fisher's exact test was used for evaluation of tumor incidence, Student's t test for two-group comparisons, and one-way ANOVA for multiple-group comparisons. Values were expressed as mean ± SD. P<0.05 was considered statistically significant.

## SUPPLEMENTARY MATERIALS FIGURES AND TABLES











## Abbreviations

4HAQO: 4-hydroxyaminoquinoline 1-oxide
4NQO: 4-nitroquinoline 1-oxide
8OHdG: 8-oxo-2′-deoxyguanosine
CP: canonical pathway
EIA: enzyme immunoassay
GCLC: glutamate-cysteine ligase catalytic subunit
GSA: gene set analysis
GSH: glutathione
GST: glutathione transferase
HO1: heme oxygenase 1
IHC: immunohistochemical staining
IOD: integrated optical density
MSB: 4-methyl sulfinylbutylamine
MRP: multidrug resistance protein
MT1: metallothionein 1
NQO1: NAD(P)H dehydrogenase [quinone] 1
NRF2: nuclear factor, erythroid 2-like 2
OLK: oral leukoplakia
ROS: reactive oxygen species
SAM: significance analysis of microarrays
SFN: sulforaphane
SCC: squamous cell carcinoma
SNV: single nucleotide variant
SOD: superoxide dismutase.
