# Supplementary material for: Chemoprevention of oxidative stress-associated oral carcinogenesis by sulforaphane depends on NRF2 and the isothiocyanate moiety
Source: Oncotarget. 2016 Jul 15;7(33):53502–14. doi: 10.18632/oncotarget.10609 (PMC5288201; doi:10.18632/oncotarget.10609)
Supplement: Supplementary file 1 [file oncotarget-07-53502-s001.pdf]

Chemoprevention of oxidative stress-associated oral carcinogenesis by sulforaphane depends on NRF2 and the isothiocyanate moiety

SUPPLEMENTARY DATA

To a stirred solution of 4-aminobutan-1-ol (**1**) (1g, 11.25 mmol) in THF (15 mL) was added $(Boc)_2O$ (2.58g, 11.8 mmol, 1.05eq) at r.t., and the mixture was stirred at r.t. for 1h. The solution was concentrated to dryness, and the oily residue was purified by flash column chromatography (petroleum ether: EtOAc=1:1) to give *tert*-butyl (4-hydroxybutyl)carbamate (**2**) as colorless oil that solidified to a white solid on standing (2.2g, 99% yield). ^{1}H NMR (500 MHz, $CDCl_3$): δ1.37 (s, 9H), 1.50 (m, 4H), 3.05 (m, 2H), 3.56 (m, 2H); ^{13}C NMR (125 MHz, $CDCl_3$): δ26.71, 28.59, 29.87, 40.48, 62.23, 79.30, 156.47.

MsCl (1.08 mL, 13.92 mmol, 1.2 eq) was added dropwise over 5 min to an ice-cold stirred solution of **2** (2.2g, 11.6 mmol) and Et_3N (2.42 mL, 17.4 mmol, 1.5 eq) in anhydrous CH_2Cl_2 (20 mL). The mixture was stirred at 0 °C for 30 min, then allowed to warm to r.t. with stirring for 1h to give a yellow suspension. The suspension was filtered off and the filter cake was rinsed with CH_2Cl_2 (5 mL). The combined filtrate was washed with H_2O (10 mL) and brine (10 mL), dried (Na_2SO_4), and concentrated to give mesylate **3** (~2.98g) as a colorless oil that was used in the next step without further purification.

A solution of the crude **3** (~2g, 7.5 mmol) in anhydrous DMF (8 mL) was added dropwise to a stirred mixture of NaSMe (764 mg, 11.3 mmol, 1.5 eq) in anhydrous DMF (4 mL) at 0 °C within 12 min to give a thick yellow suspension. The mixture was stirred for 10 min, the ice-bath was removed, and stirring was continued for an additional 1h to give a gray-yellow suspension. The mixture was extracted with EtOAc (3×20 mL) and the combined extracts were washed with H_2O (20 mL) and brine (20 mL) then dried (Na_2SO_4). The crude product was purified by flash column chromatography (petroleum ether: EtOAc=4:1) to give *tert*-butyl [4-(methylsulfanyl) butyl]carbamate (**4**) as light-yellow oil (1.49g, 90% yield based on **2**). ^{1}H NMR (500 MHz, $CDCl_3$): δ1.39 (s, 9H), 1.54 (m, 4H), 2.04 (s, 3H), 2.46 (t, J=7.2Hz, 2H), 3.08 (m, 2H); ^{13}C NMR (125 MHz, $CDCl_3$): δ15.65, 26.48, 28.60, 29.40, 34.04, 40.32, 79.24, 156.18.

A solution of **4** (2.1g, 9.62 mmol) in CH_2Cl_2 (15 mL) was chilled to -10 °C in an ice-salt bath. A solution of 85% *m*-CPBA (1.97g, 9.62 mmol) in CH_2Cl_2 (15 mL) was added dropwise over 20 min while the internal temperature was kept below 0 °C. When the addition was completed, the mixture was stirred at 0 °C for 1h and then the resulting light-yellow suspension was treated with saturated aq. $NaHCO_3$ (20 mL). The organic layer was separated and the aqueous layers were washed with brine (20 mL), dried (Na_2SO_4) and concentrated by a rotary evaporator. The crude product was purified by flash column chromatography (DCM:MeOH=50:1) to give 4-(*tert*-butoxycarbonyl)butyl methyl sulfoxide (**5**) as light-yellow oil (2.18g, 96% yield). ^{1}H NMR (500 MHz, $CDCl_3$): δ1.83 (m, 4H), 2.53 (s, 3H), 2.67 (t, J=6.9Hz, 2H), 3.54 (t, J=5.4Hz, 2H); ^{13}C NMR (125 MHz, $CDCl_3$): δ20.27, 29.18, 38.94, 44.86, 53.66, 130.93.

A solution of **5** (1.5g) and formic acid (9 mL) was stirred at r.t. for 2h. The excess formic acid was removed at r.t. by a rotary evaporator to afford 4-(methylsulfinyl) butylamine (**MSB)** as a light-yellow oil (1.0g, 86% yield). ^{1}H NMR (300 MHz, CD_3OD): δ1.83 (m, 4H), 2.66 (s, 3H), 2.85 (m, 2H), 2.98 (t, J=7.2 Hz, 2H); ^{13}C NMR (300 MHz, DMSO-d_6): δ19.33, 26.33, 37.98, 38.47, 52.41; MS (ESI): m/z 136 $[M+1]^+$.

Chemical Synthesis of MSB

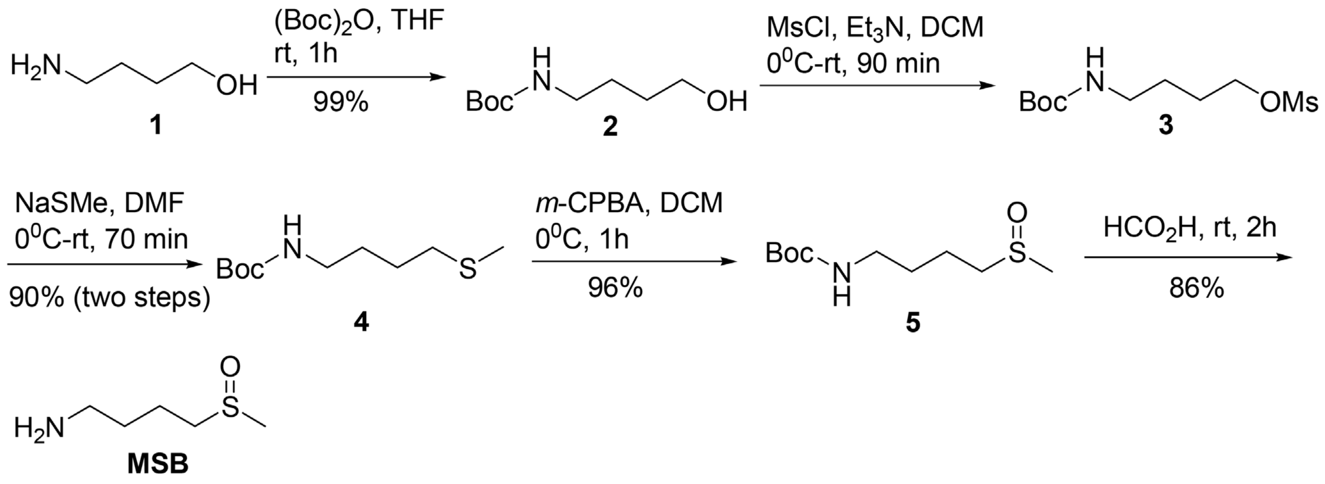

Supplementary File. Method for chemical synthesis of MSB.

Supplementary Excel 1: SAM analysis of gene array data by comparing control and 4NQO-treated mouse tongue. On the spreadsheet named "SAM output", 32 genes are up-regulated by 4NQO treatment, and 1 gene down-regulated.

See Supplementary File 1

Supplementary Excel 2: GSA analysis of gene array data using curated gene sets according to canonical pathways. On the spreadsheet named "SAM outpout", 58 gene sets are up-regulated by 4NQO treatment and 34 gene sets down-regulated.

See Supplementary File 2

Supplementary Excel 3: GSA analysis of gene array data using an NRF2-regulated gene set. Spreadsheet "NRF2-regulated gene set" contains a gene list obtained from our previous study on $Nrf2^{-/-}$ mouse esophagus [60].

See Supplementary File 3

Supplementary Excel 4: Single nucleotide variants in 4NQO-treated mouse tongue samples identified by whole exome sequencing. Spreadsheet "normal vs 4NQO1" shows mutations in one mouse tongue treated with 4NQO, and spreadsheet "normal vs 4NQO2" shows mutations in one mouse tongue treated with 4NQO and ethanol.

See Supplementary File 4
